# Effects of vitamin D_3_ release from 3D printed calcium phosphate scaffolds on osteoblast and osteoclast cell proliferation for bone tissue engineering

**DOI:** 10.1039/c9ra06630f

**Published:** 2019-10-29

**Authors:** Ashley A. Vu, Susmita Bose

**Affiliations:** W. M. Keck Biomedical Materials Research Laboratory, School of Mechanical and Materials Engineering, Washington State University, Pullman, Washington 99164, USA.

## Abstract

Vitamin D_3_ is a hydrophobic micronutrient and is known for inhibiting osteoclastic bone resorption *in vivo via* suppression of the Receptor Activator of Nuclear factor-Kappa B (RANK ligand) expression in osteoblasts. Although vitamin D is well-known for its promotion in bone health, little is known on its effects directly on bone cells. The objective of this study was to understand the effects of vitamin D_3_ release from 3D printed calcium phosphate scaffolds towards bone cell proliferation. In this study, cholecalciferol, a common intake form of vitamin D_3_, was successfully able to release from the scaffold matrix *via* the use of polyethylene glycol. Results showed a decrease in osteoclast resorption pits and healthier osteoblast cellular morphology compared to the control. Additively manufactured tricalcium phosphate scaffolds with designed porosity were loaded with vitamin D_3_ and showed controlled release profiles in phosphate buffer and acetate buffer solutions. The release kinetics of vitamin D_3_ from calcium phosphate scaffolds enabling osteoblast proliferation and inhibiting osteoclastic resorption can enhance healing for low load bearing applications for bone defects or permeate voids left by tumor resection.

## 1. Introduction

**Fig. 1:**
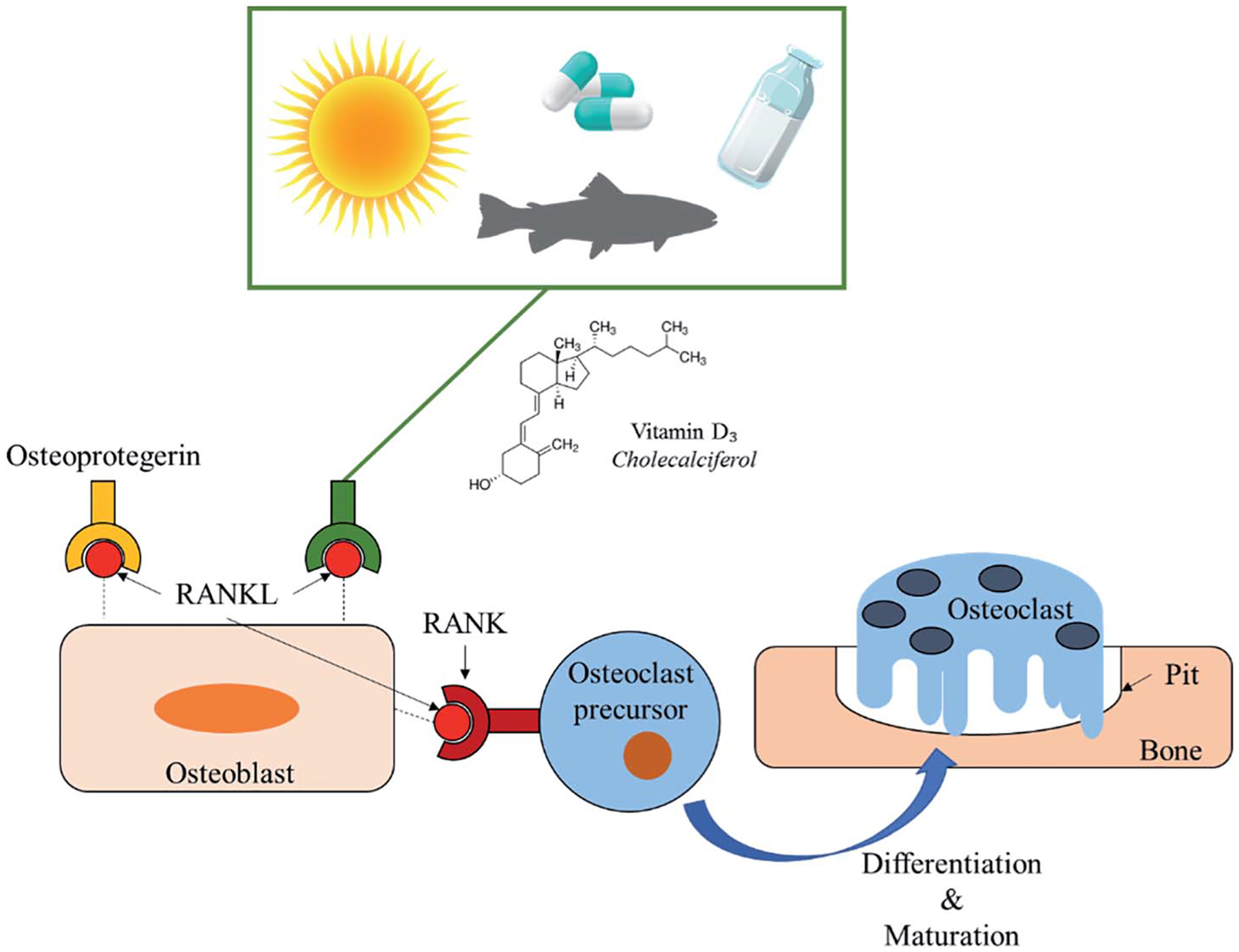
Possible effect of vitamin D_3_ on osteoclastic resorption: osteoblasts express RANKL, an apoptosis regulatory gene, which binds to RANK receptors on osteoclast precursors or to osteoprotegerin receptors. Vitamin D_3_ (cholecalciferol) derived *via* skin absorption from sun, supplements, and diet also can act as a receptor for RANKL causing inhibition of osteoclast precursors from competitive binding and differentiating into mature osteoclasts, eventually reducing osteoclastic resorption.

More than 8.9 million fractures occur annually due to osteoporosis.^1^ Osteoporosis is caused by many factors including a deficiency of calcium or vitamin D_3._^2^ Risk of vitamin D deficiency occurs from little sun exposure and/or poor dietary intake.^3^ Low vitamin D levels, hypovitaminosis D, can be associated with higher secretion of parathyroid hormone, increased bone turnover, osteoporosis, osteomalacia, and higher risk for hip and other fractures.^4,5^ Vitamin D_3_ is a hydrophobic micronutrient and is known for inhibiting osteoclastic bone resorption *in vivo via* suppression of the Receptor Activator of Nuclear factor-Kappa B (RANK ligand) expression in osteoblasts (Fig. 1).^6,7^ The recommended daily intake of vitamin D_3_ varies from 10 to 100 μg per day. Analogs of vitamin D_3_ such as alfa-calcidol have been used for osteoporosis since 1983 as therapeutic agents to increase bone mineral density and reduce incidences of bone fracture.^8–10^ Vitamin D_3_ and its receptors have also been reported to modulate lymphocyte and macrophage functions,^11^ impact the immune system,^12,13^ lower risk for cardiovascular mortality in haemodialysis patients,^14^ and even provide potential anticancer properties.^15,16^

The objective of this study was to understand the effects of vitamin D_3_ release from 3D printed calcium phosphate scaffolds towards bone cell proliferation.† To the best of the authors’ knowledge, the effects of vitamin D_3_ on osteoblast and osteoclast bone cell proliferation for bone tissue engineering applications using bioresorbable scaffolds has yet to be explored. Furthermore, limited studies have been done regarding the effects of cholecalciferol *in vitro*. One study successfully provided local delivery of cholecalciferol within hydroxyapatite nano-particles through the use of poly(d,l-lactide-*co*-glycolide).^17^ Another study utilized complex nanoparticles for vitamin D_3_ encapsulation as well as controlled release in simulated gastric fluid and simulated intestinal condition.^18^ Other works have shown benefits of vitamin D_3_ and its analogs within the body to aid in osteoporotic fractures through oral digestion.^19,20^

The hypothesis of this study was vitamin D_3_ would lower osteoclast resorption and show no cytotoxic effects towards osteoblast cells. The hydrophobic nature of vitamin D_3_ makes it difficult to release in an aqueous solution such as the physiological environment inside the body. Vitamin D_3_ enhanced hydrophilicity by using polyethylene glycol (PEG).^21^ PEG has been known for its use in medicine due to its various abilities such as facilitating drug absorption.^22^ Not only is PEG biocompatible, non-immunogenic, and highly soluble in water, it is also approved by the Food and Drug Administration (FDA).^23^ Polycaprolactone (PCL) is a biodegradable polymer used for drug delivery applications to modulate release kinetics.^24,25^ PCL is also FDA approved, biocompatible, relatively low in cost, and easy to process. In this study, a combination of both PEG and PCL was utilized for loading vitamin D_3_ into bone tissue engineering scaffolds. Hydroxyapatite (HA) and tricalcium phosphate (TCP) have excellent biocompatibility, biodegradability, and high osseointegration making them widely used materials for bone tissue engineering.^26,27^ TCP scaffolds also have the ability to be an effective, local drug delivery system when used as a bioresorbable implant.^28,29^ Both were utilized *in vitro* with osteoclast and human fetal osteoblast (hFOB) cell lines. Porous TCP scaffolds were produced using powder bed additive manufacturing to investigate *in vitro* release kinetics of vitamin D_3._ These advanced manufactured scaffolds provide the ability for patient specific implants with complex structures. The results from this study could be incorporated in low load bearing applications to provide local vitamin D_3_ drug delivery for bone defects or voids caused by tumor resection.

## 2. Materials and methods

### 2.1 Vitamin D_3_/polymer loading

Vitamin D_3_ (cholecalciferol, Alfa Aesar, Ward Hill, MA) was dissolved in ethanol at a concentration of 10 mg mL^−1^. Polymer solution of PCL (*M*_w_ = 14 000, Sigma-Aldrich, MO) and PEG (*M*_w_ = 8000, Sigma-Aldrich, MO) at a 65 : 35 molar ratio was prepared. Polymer mix was dissolved in acetone at 5 wt% of PCL/PEG. Drug and polymer solutions were mixed together at a ratio of 50 : 50 Any processing with vitamin D_3_ was done in the dark due to its sensitivity to light.

### 2.2 Effects of vitamin D_3_ *in vitro* on osteoclast and osteoblast cells

Samples used in the osteoclast cell culture study were HA (Monsanto, USA) discs and osteoblast cell culture study were synthesized TCP discs. Specific synthesis methods can be found in detail in previous works.^27^ Both discs were 12 mm diameter and 2.5 mm height uniaxially pressed at 165 MPa for 2 min each and sintered at 1250 °C for 2 h in a muffle furnace. All samples used in the study were sterilized in an autoclave for 1 h at 121 °C prior to any drug and polymer loading. All samples with vitamin D_3_ contained 20 μg of drug.

### 2.3 Osteoclast cell protocol and resorption pit assay

Osteoclast cells (THP1 monocytes, ATCC, Manassas, VA) were seeded onto samples with a density of 25 000 cells per sample. Growth media was comprised of Roswell Park Memorial Institute (RPMI)-1640, 0.05 mM 2-mercaptoethanol, and 10% FBS. Differentiation media was comprised of 40 ng mL^−1^ phorbol 12-myristate 13-acetate (PMA) (Sigma Aldrich, St. Louis, MO) and 10 ng mL^−1^ RANKL with RPMI-1640 and FBS. All cultures were kept at 37 °C under an atmosphere of 5% CO_2_ and the medium was changed every 2–3 days throughout the experiment. A timepoint of 10 days was utilized to assess resorption pits. Samples were ultrasonicated in 1 mL of 1 M NaCl solution mixed with 0.2% Triton X-100. Samples were rinsed, dehydrated, and gold coated prior to Scanning Electron Microscopy (SEM) imaging. Protocol for dehydration can be found in other work.^25^

### 2.4 Osteoblast cell protocol and viability assays

Human fetal osteoblast cells (hFOB 1.19, ATCC, Manassas, VA) were seeded onto samples with a density of 79 200 cells per sample. Osteoblast cell viability and morphology were analyzed using MTT (3-(4,5-dimethylthiazol-2-yl)-2,5-diphenyltetrazolium bromide) assay and SEM, respectively, with timepoints of 3, 7, and 11 days. Specific protocols can be referenced in other work with the exception of using ATCC recommended growth medium.^26^

### 2.5 3D printed scaffold fabrication and characterization

Synthesized TCP scaffolds with 500 μm designed pore size were fabricated (6.7 mm diameter × 11 mm height) in a 3D powder bed printer (ExOne, Irwin, PA, USA). Fabrication and characterization methods including compressive strength testing using a screw-driven universal testing machine equipped with a load cell can be referenced in previous work.^27,29^

### 2.6 Vitamin D_3_ *in vitro* release study

Scaffolds were immersed in each buffer solution at 4 mL each timepoint. All samples were kept in a shaker at 37 °C with 150 rpm of constant shaking. Buffer solutions were changed periodically with fresh buffer added. Release was stopped after 7 days in ABS to eliminate erroneous data due to scaffold degradation. The concentration of vitamin D_3_ release at each timepoint was determined using a Biotek Synergy 2 SLFPTAD microplate reader (Biotek, Winooski, VT, USA) at an absorbance value of 260 nm wavelength. A standard curve was created using known concentrations of vitamin D_3_ in buffer solutions. A control of PCL/PEG loaded was subtracted from VD_3_/PCL/PEG release to quantify each timepoint.

### 2.7 Statistical analysis

All compositions in each scaffold characterization test, compositions in the release study, and MTT assay were analyzed in triplicate with each measurement performed in triplicate. Data is presented as mean ± standard deviation. Comparative analysis in MTT results showed no statistical differences at a *p* < 0.05 confidence level.

## 3 Results and discussion

**Fig. 2:**
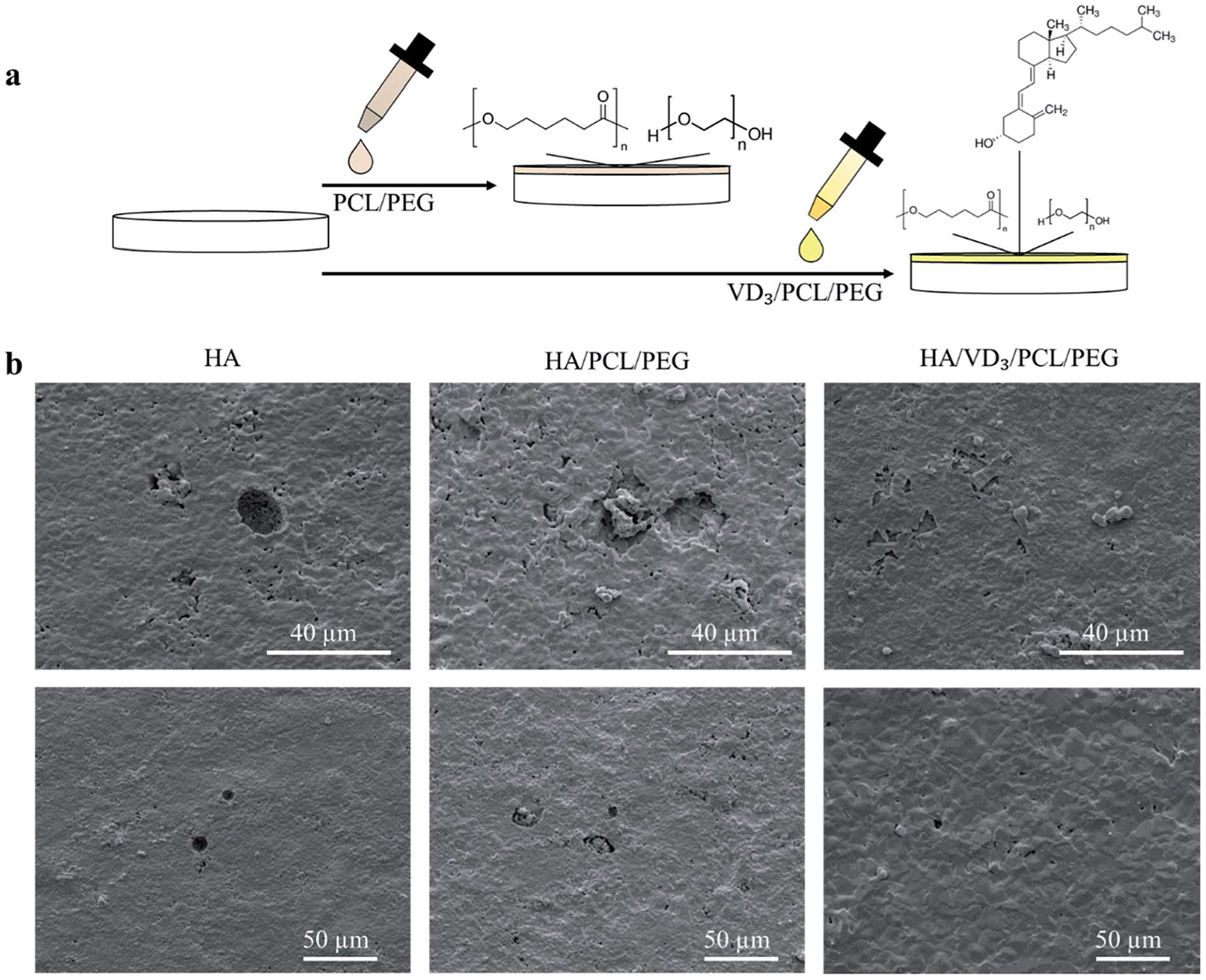
Effects of vitamin D_3_ release on osteoclast cell proliferation. (a) Schematic of sample preparation. (b) Osteoclast resorption pit assay showing clear resorption pits on HA and HA loaded with PCL/PEG samples however little to no resorption pits could be found on samples loaded with vitamin D_3_ indicating reduced osteoclast activity.

To assess bone cell materials interaction, cell cultures were performed with osteoblast cells and osteoclast cells separately followed by scanning electron microscopy (SEM) to assess morphology. The SEM images following osteoclast culture showed resorption pits of 20–30 μm size all over the surface of control HA and HA samples loaded with PCL/PEG (Fig. 2).

**Fig. 3:**
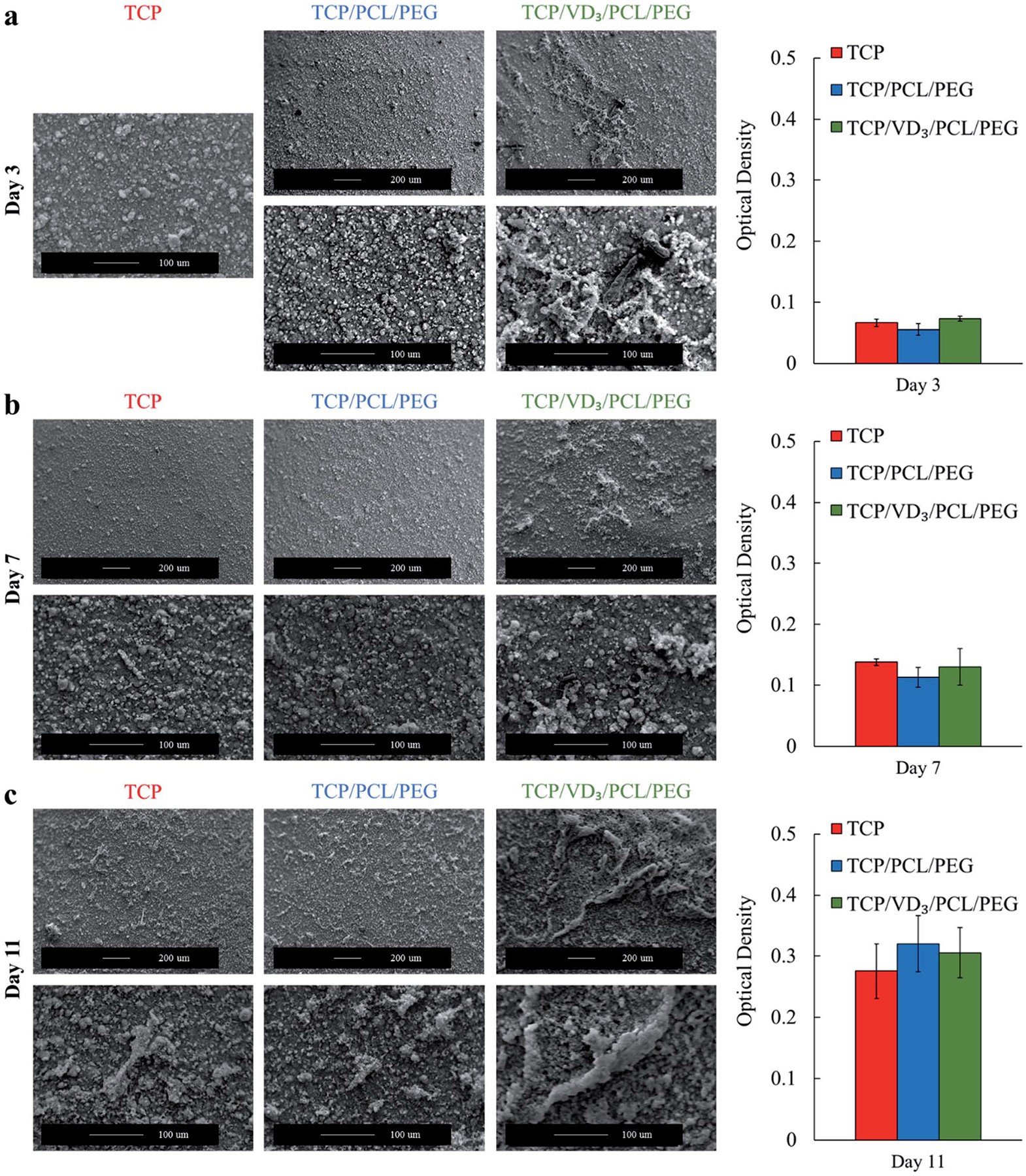
MTT assay optical cell density and SEM images with hFOB after (a) 3, (b) 7, (c) 11 days of culture showing no cytotoxic effects with PCL/PEG or VD_3_/PCL/PEG loading. Highest cell morphology seen in 11 days of culture as well as cell morphology seen in all timepoints with vitamin D_3_ but only apatite formation in day 3 and day 7 with TCP and TCP/PCL/PEG samples.

Resorption pits were unable to be identified on samples loaded with vitamin D_3_/PCL/PEG indicating reduced osteoclastic activity. The use of vitamin D_3_ also showed no cytotoxic effects towards hFOB cells with higher cell coverage and healthier cellular morphology compared to control. MTT assay showed no statistically significant differences in cell viability for samples with PCL/PEG and VD_3_/PCL/PEG compared to control TCP (Fig. 3). Cytotoxicity is determined by a threshold of 70% viability in the first 24 h and neither PCL/PEG nor VD_3_/PCL/PEG loaded TCP samples showed cytotoxic effects in all timepoints.^30^ The morphology of the hFOB cells after 3 days of culture (Fig. 3a) showed mainly apatite formation with only the presence of cells on samples with vitamin D_3_. After 7 days of culture (Fig. 3b), the trend continued of cells being more prominent on samples loaded with vitamin D_3_. After 11 days of culture (Fig. 3c), cells were present on all compositions with apatite formation on top and vitamin D_3_ showed highest coverage of cells.

**Fig. 4:**
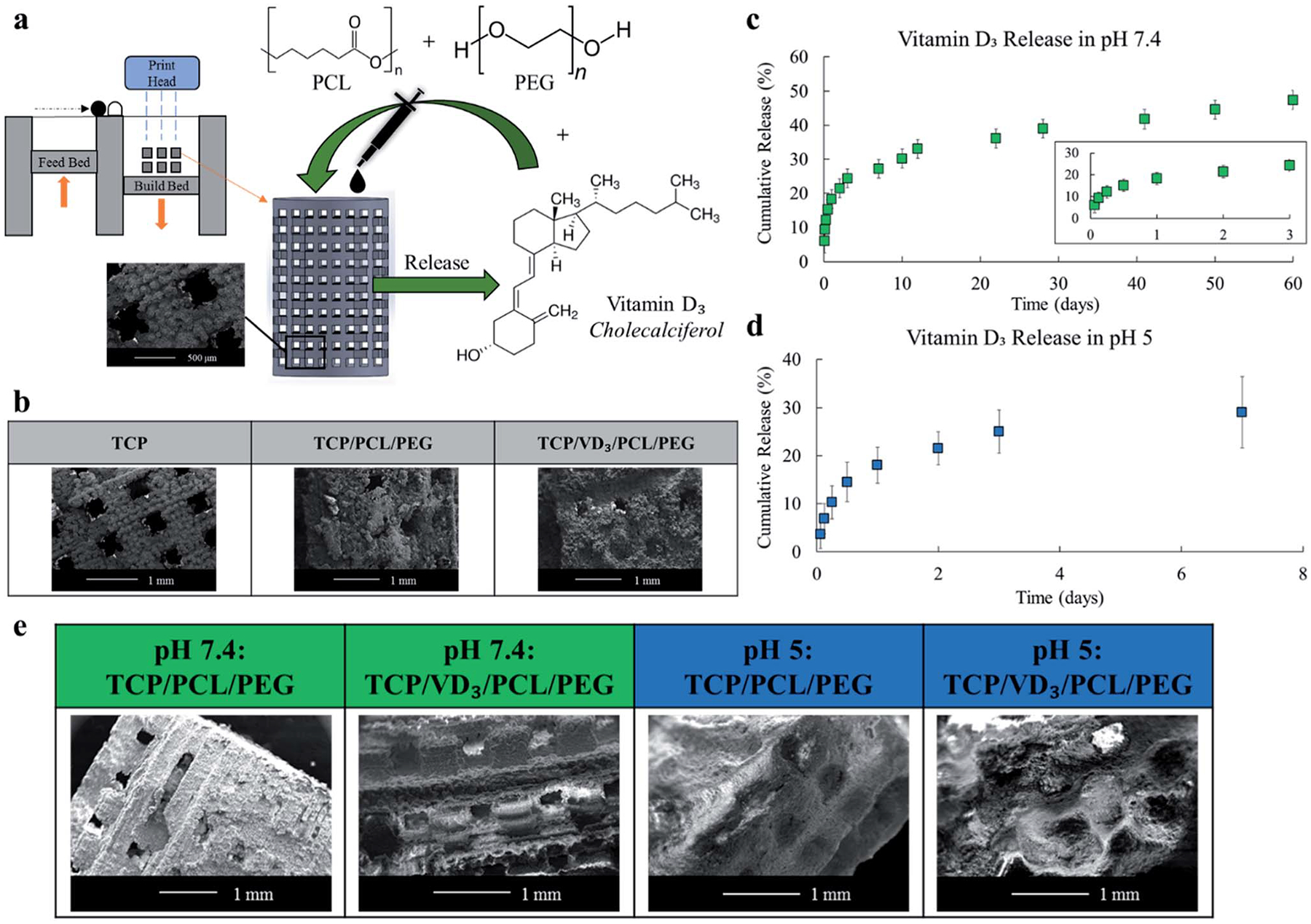
Release profiles and degradation characteristics of vitamin D_3_ loading. (a) Schematic representation of 3D printed porous TCP scaffolds with 500 μm designed pores and loading with VD_3_/PCL/PEG to achieve release of VD_3_. (b) Pore size of scaffolds range from 325 to 380 μm. Comparable degradation is seen between both PCL/PEG and VD_3_/PCL/PEG. (c and d) Vitamin D_3_ release from VD_3_/PCL/PEG loaded 500 μm-3DP scaffolds in PBS over 60 days and ABS over 7 days showed faster release in ABS. (e) Degradation of scaffolds between vitamin D_3_ loaded and control with polymer was comparable both before release study and after.

Porous scaffolds are structures that allow for the interaction of cells and extracellular matrices. Scaffolds also provide structural support for growing cells especially in circumstances involving bone defects and voids left by tumor resection. Manufacturing scaffolds using 3D printing allows for site specificity such as oral and maxillofacial defects (Fig. 4a). Pore sizes must be large enough for cell mobility, but scaffolds must also have enough structural integrity for use within the body. The 500 μm-3DP porous scaffolds used in this study have pore sizes ranging from 325 to 380 μm post sintering as assessed through SEM images (Fig. 4b). Pore sizes should be between 50 to 500 μm (ASTM F2150) where greater than 300 μm is most recommended. These scaffolds had a compressive strength of 4.0 ± 1.54 MPa. Cancellous bone, the spongy bone where these scaffolds would mostly interact with, has a compressive strength ranging from 0.22 up to 10.44 MPa.^31^ Bulk density, volume fraction open porosity, and relative density to TCP of the scaffolds were assessed to be 0.6 ± 0.01 g cm^−3^, 56.4 ± 0.31%, and 26.9 ± 0.01% respectively.

Scaffolds were loaded with 500 μg of vitamin D_3_ (500 μm-3DP) to investigate release kinetics in phosphate buffer solution (PBS, pH 7.4) to mimic the physiological environment and acetate buffer solution (ABS, pH 5) to mimic the micro-acidic environment post injury (Fig. 4c and d).^32,33^ The release of vitamin D_3_ depends significantly through the physicochemical interactions between the vitamin and the polymer system. Through the use of PEG, particles will aggregate *via* steric stabilization and increase stability during storage and application of the system.^34^

Additionally, PEG is a non-ionic hydrophilic polymer with stealth behavior; long circulating nanocarriers that avoid opsonization.^35^ The hydrophobic–hydrophilic interaction of vitamin D_3_ and PEG enables the release of vitamin D_3_ due to the more favorable solubility of the vitamin into the media compared to the binding within the scaffold matrix. With only PEG, vitamin D_3_ would show burst release in early timepoints, a phenomenon indicative in many drug releases.^36–38^ Burst release can be both pharmacologically dangerous, inefficient for long term delivery, and economically unfavorable. Minimal or eliminated burst release and prolonged release can be achieved using polymers such as PCL; a biodegradable polymer used to modulate release kinetics.^23–25,39–41^ PCL is degradable through hydrolysis of the ester linkages which can occur in physiological conditions.^42^ The hydrophobic properties of PCL limited the potentially higher burst release of vitamin D_3_ and enabled a more controlled, prolonged release over time. Surface degradation could be seen in all samples released in either buffer solution (Fig. 4e). Degradation of scaffolds with polymer and drug/polymer loading was comparable prior and following buffer release indicating drug loading did not worsen surface degradation (Fig. 4b and e). Scaffolds released in ABS showed heavy microstructural degradation compared to scaffolds released in PBS, as expected due to the higher acidity rapidly breaking down the scaffold matrix.

## 4. Conclusion

Vitamin D_3_ in the form of cholecalciferol showed reduced osteoclastic activity alongside no cytotoxicity effects towards osteoblast cells. Little to no resorption pits were identified with vitamin D_3_/PCL/PEG loaded HA samples compared to control and polymer loaded indicating a reduction in osteoclast activity. Scanning electron microscopy images showed more osteoblast cell morphology on samples with vitamin D_3_ compared to control TCP and TCP with PCL/PEG as well as no cytotoxicity assessed through MTT assay. A controlled release of vitamin D_3_ from additively manufactured porous TCP scaffolds with interconnected porosity was effectively achieved using a mixed polymer system of PCL and PEG. These results indicate vitamin D_3_ can be successfully released from TCP porous scaffolds to provide an effective, local drug delivery system for bone tissue engineering low load bearing applications and provide aid for bone defects or permeate voids left by tumor resection.

## Footnotes

† Vitamin D3 (cholecalciferol) was obtained from Alfa Aesar, Ward Hill, MA.
